# Which MR sequences should we use for the reliable detection and localization of bone marrow edema in spondyloarthritis?

**DOI:** 10.1007/s11547-017-0778-y

**Published:** 2017-06-07

**Authors:** Chiara Giraudo, Michael Weber, Antonia Puchner, Johannes Grisar, Franz Kainberger, Claudia Schueller-Weidekamm

**Affiliations:** 1 Division of Neuroradiology and Musculoskeletal Radiology, Department of Biomedical Imaging and Image-guided Therapy, Medical University of Vienna, Waehringer Guertel 18-20, 1090 Vienna, Austria; 2 Division of Rheumatology, Department of Internal Medicine III, Medical University of Vienna, Vienna, Austria; 3 Second Department of Internal Medicine with Rheumatology/Osteology and Gastroenterology, KH Barmherzige Schwestern (St. Vincent Hospital), Vienna, Austria; 4 Karl Landsteiner Institute for Gastroenterology and Rheumatology, St. Pölten, Austria

**Keywords:** MRI, Rheumatology, Sacroiliitis, Spondyloarthritis

## Abstract

**Objectives:**

To assess the diagnostic confidence in detecting and localizing areas of bone marrow edema in the sacroiliac joint of patients with suspected spondyloarthritis using a single-plane method and comparing it with multiplanar unenhanced and enhanced methods.

**Materials and methods:**

Patients with clinical suspicion of spondyloarthritis undergoing an MRI of the sacroiliac joint were included in this retrospective study. To assess sacroiliitis, three methods were applied: single-plane (i.e., para-coronal STIR alone), multiplanar unenhanced (i.e., para-coronal STIR and para-axial PD-fs), and multiplanar enhanced method (i.e., para-coronal and para-axial post-contrast T1-fs). Two 4-point scales were used to evaluate, respectively, the diagnostic confidence in detection and localization of bone marrow edema. The distribution of certain and uncertain rating according to signal intensity and size of the lesions was also calculated.

**Results:**

Seventy-four patients met the inclusion criteria. Both multiplanar methods increased the diagnostic confidence in detection (*p* < 0.001) and localization (*p* < 0.001) of sacroiliitis; no significant difference occurred between the multiplanar unenhanced and enhanced methods (*p* = 0.405 and *p* = 1.00, respectively, for detection and localization). A statistically significant difference between the distributions of certain and uncertain rating for detection based on the size and signal intensity of each lesion emerged (*p* = 0.006 and *p* < 0.001, respectively), whereas no statistically significant difference occurred for the confidence of localization (*p* = 0.452 and *p* = 0.694, respectively).

**Conclusions:**

The multiplanar methods increased the diagnostic confidence in detection and localization of sacroiliitis. The absence of a significant difference between the proposed unenhanced and enhanced methods suggests that contrast medium is not mandatory for the detection of sacroiliitis.

## Introduction

Radiographic sacroiliitis had a primary role for the diagnosis, classification, and monitoring of patients with spondyloarthritis (SpA) since its introduction among the Rome classification criteria in 1961 and the modified NY criteria in 1984 [1–3].

In 2009, the Assessment of Spondyloarthritis International Society (ASAS) [4] listed magnetic resonance imaging (MRI) findings of active inflammatory lesions among the diagnostic criteria for SpA, and, since then, much effort has been devoted to optimizing a dedicated MRI protocol for this category of patients. Thus, several studies have focused not only on the role of STIR and post-contrast sequences (i.e., the main sequences recommended by the ASAS), with the aim of evaluating the necessity for contrast medium (CM) application [5–8], but also on the utility of a multiplanar approach to accurately investigate a joint as complex as the sacroiliac joint (SIJ) [9–12]. A recently published study showed good diagnostic performance for para-axial proton density fat-sat sequences (PD-fs) for both the acute and chronic findings associated with SpA [13]. However, to date, to the best of our knowledge, no studies have been performed to assess how a multiplanar non-enhanced method, including STIR and para-axial PD-fs, might affect the detection and localization of areas of bone marrow edema (BME).

Thus, the aim of this study was to evaluate the diagnostic confidence in detecting and localizing BME in patients with clinical findings suggestive of SpA, using para-coronal STIR alone, and comparing it with the diagnostic confidence using a multiplanar method without contrast application, which included para-coronal STIR and para-axial proton density fat-saturated sequences, and with a multiplanar contrast-enhanced method.

## Materials and methods

### Patients and study design

Patients with clinical findings suggestive of SpA according to ASAS guidelines [4], referring to our tertiary center for diagnostic assessment with MRI of the SIJ between September 2013 and June 2015 were included in this retrospective, Institutional Review Board approved study. As radiologic inclusion criteria, the MRI examinations had to be conducted in our Institution applying a standardized protocol including para-coronal STIR, para-axial PD-fs, para-coronal T1, para-axial and para-coronal T1-fs post-contrast administration, aiming to avoid any bias due to different devices and/or protocols.

### Image analysis

To assess the diagnostic confidence in the identification and localization of BME, three methods were applied: a single-plane method (i.e., using para-coronal STIR alone); a multiplanar method without CM (i.e., including para-coronal STIR and para-axial PD-fs); and a postcontrast multiplanar method (i.e., including para-coronal and para-axial post-contrast T1-fs) (Table 1).

**Table 1:** MRI methods applied for the detection and localization of the areas of bone marrow edema affecting the sacroiliac joint of patients with clinical findings suggestive of spondyloarthritis

Method	Sequences^a^	Sequences parameters^b^
		(TR/TE, FOV, matrix, slice thickness)
Single plane method	Para-coronal STIR	4263/75, 230 × 230, 576 × 382, 3 mm
Multiplanar unenhanced method	Para-coronal STIR	4263/75, 230 × 230, 576 × 382, 3 mm
	Para-axial PD-fs	8140/30, 350 × 254, 576 × 382, 3 mm
Multiplanar enhanced method	Para-coronal T1-fs	597/10, 260 × 260, 322 × 284, 3 mm
	Para-axial T1-fs	690/8, 250 × 250, 276 × 236, 3 mm

^a^Para-axial and para-coronal planes were performed respectively, parallel and perpendicular to the upper endplate of the first sacral vertebral body

^b^All examinations were conducted with a 3T MR (Philips Achieva; Philips Medical Systems, Best, The Netherlands) using a body-array coil, our clinical protocol for SpA includes also a TSE-T1w coronal sequence, not mentioned above because the T1w datasets were not used in the hereby presented study for investigating areas of bone marrow edema

Two radiologists (C.SW, C.G.) with 13 and 5 years of experience in musculoskeletal radiology, blinded to patients’ data (i.e., clinical parameters, patient’s history, findings of other imaging modalities), analyzed each set of images in consensus, according to ASAS guidelines. Each patient has been assessed using the three above-mentioned methods (i.e., single plane, multiplanar unenhanced and multiplanar enhanced) and applying an interval of 4 weeks among the respective analyses, aiming thus to reduce any potential imaging readers bias.

### Diagnostic confidence in detection of BME

**Fig. 1:**
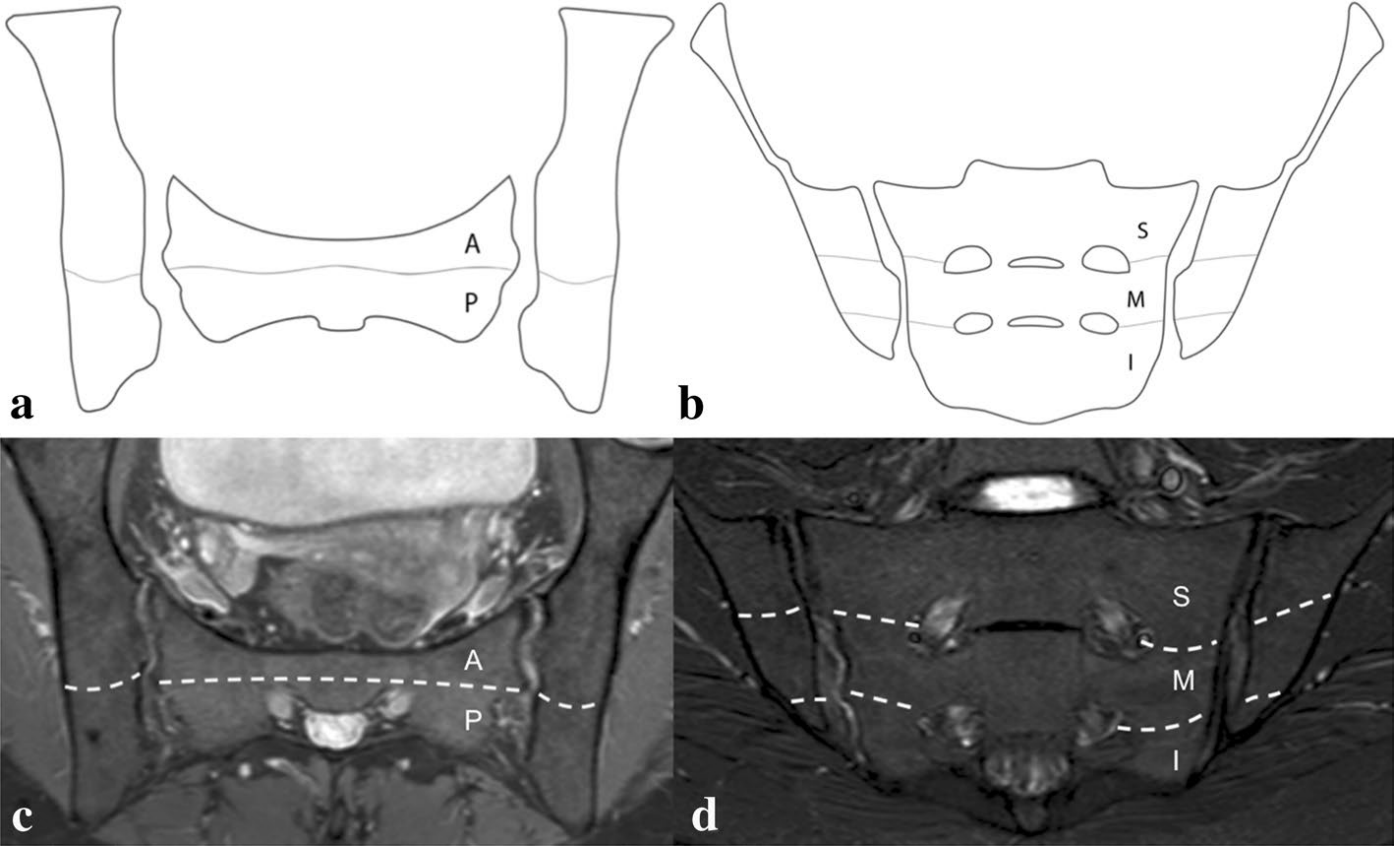
Drawing of the sacroiliac joint demonstrating the anatomical subdivision (i.e., *light gray lines*) applied to assess the joint on the para-axial (**a**) and para-coronal plane (**b**) (i.e., *A* anterior, *P* posterior, *S* superior, *M* middle, *I* inferior) and the corresponding subdivision on MRI images (i.e., *white dotted lines* on the para-axial PD-fs, in **c**, and para-coronal STIR, in **d**)

To assess the presence of BME, the iliac and sacral bones were partitioned on each side into the following regions: antero-superior; postero-superior; mid-anterior; mid-posterior; antero-inferior; and postero-inferior. The boundary between the superior and mid portion was traced at the level of the first sacral foramina, whereas the mid and the inferior portions were divided at the level of the second sacral foramina. The boundary between the anterior and posterior portion of each joint was established at the ligamento-cartilaginous junction [13] (Fig. 1).

A 4-point scale was used to rate the confidence in detection of BME (1 = definitely no BME; 2 = probably no BME; 3 = probably BME; 4 = definitely BME) with each method.

A patient-based assessment was also performed, and a patient was considered positive for an improvement in diagnostic confidence when he/she presented at least one region in which either the unenhanced or enhanced multiplanar method allowed a higher degree of certainty (i.e., either certain absence or presence of the lesion).

### Diagnostic confidence in localization BME

The same anatomical subdivision and the same methods used for the detection were applied for the localization of BMEs. A dedicated 4-point scale was used to rate the confidence in localization of BME (1 = unable to localize; 2 = probably unable to localize; 3 = probably able to localize; 4 = able to localize with confidence).

For the patient-based assessment, the same criteria applied for the diagnostic confidence in detection were used also for the localization.

### Signal intensity and size of BME

In addition, on STIR, the signal intensity (SI) of each BME was compared with the SI of spinal fluid and graded according to a 3-point scale (i.e., 1 = low grade; 2 = intermediate grade; 3 = high grade). The maximal cranio-caudal extension of each lesion was also assessed on STIR and classified according to a 3-point scale (<1 cm = 1; 1≥ cm <2 = 2; ≥2 cm = 3).

### Statistical analysis

Absolute frequencies and percentages are presented for categorical data.

Percentages of BME confident detection rating obtained by each method were compared using the Cochran *Q* test, followed by post hoc Bonferroni-corrected McNemar test. The ratings of each method regarding diagnostic confidence in BME localization were compared using the Friedman test, followed by post hoc Bonferroni-corrected Wilcoxon test. Additionally, aiming to test the intra-rater reliability for BME’s detection, 240 areas (i.e., ten patients, randomly selected) were evaluated a second time (i.e., after 1 year) by the same raters and the percentage of agreement for each applied method was computed.

A Chi-squared test was used to assess the distribution of certain and uncertain rating in BME detection according to SI and size of BME (i.e., evaluated on STIR); Fisher’s exact test was applied for the diagnostic confidence in localization.

The specified level of significance was *p* ≤ 0.05 for all tests. All statistical tests were performed using IBM SPSS Statistics 21.0 (IBM Corp, Armonk, NY).

## Results

Seventy-four patients (38 males and 36 females; mean age ± SD, 38.34 ± 10.88 years) who underwent an MRI of the SIJ at our Department for a clinical suspect of SpA were enrolled in this study. Thirty-seven patients (50%) turned out to be completely negative for sacroiliitis (i.e., no BMEs were evident with any of the applied methods).

### Diagnostic confidence in detection of BME

**Fig. 2:**
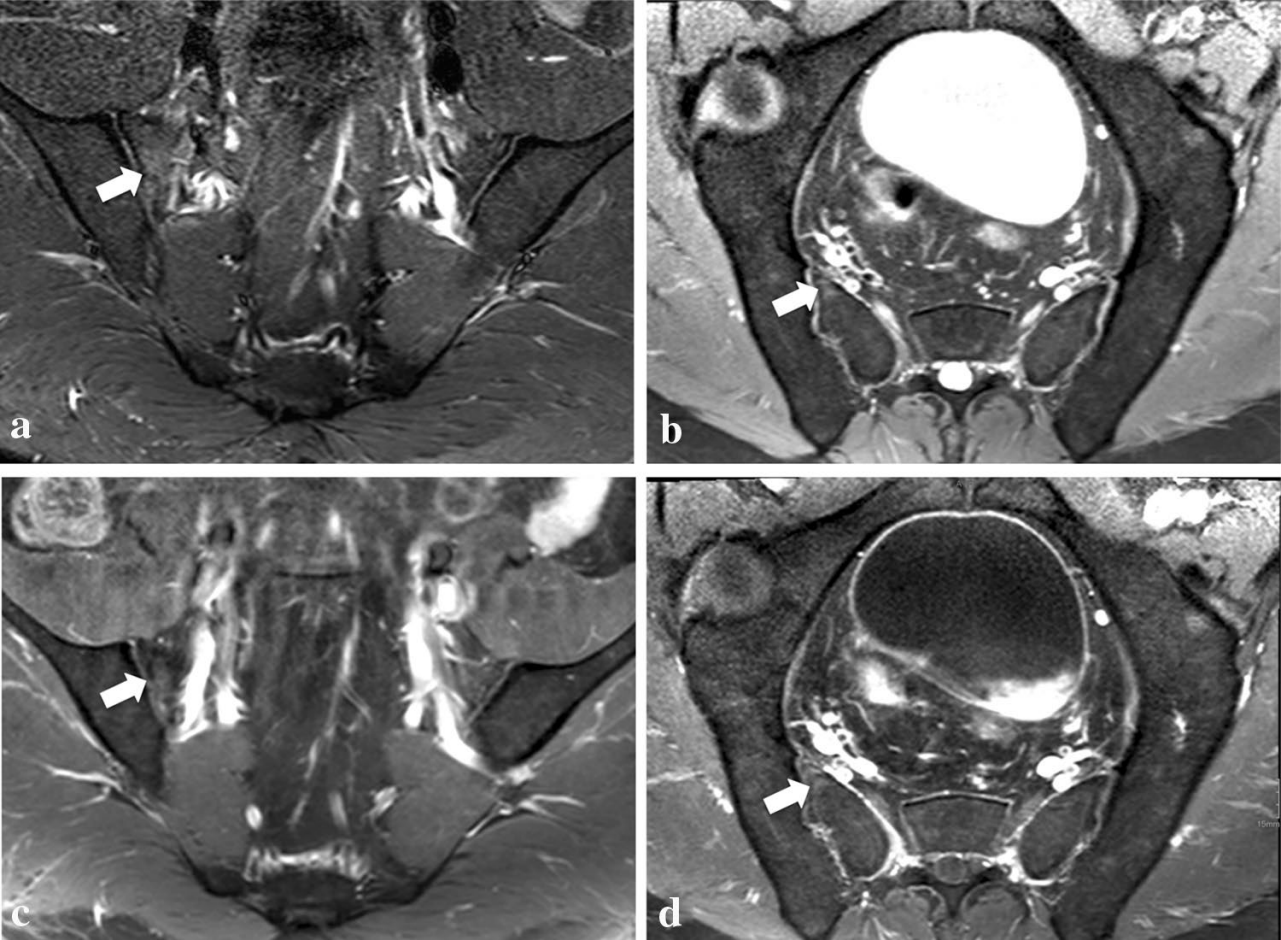
A 38-year-old male patient with clinically suspected spondyloarthritis. Low SI area of bone marrow edema in the antero-superior portion of the *right* sacrum graded as uncertain on the single-plane method (i.e., para-coronal STIR only) (**a**), and then, confidently rated with the multiplanar unenhanced method (para-coronal STIR in **a** and para-axial PD-fs in **b**) and multiplanar enhanced method (para-coronal post-contrast T1-fs in **c** and para-axial post-contrast T1-fs in **d**)

Overall, 1776 regions were analyzed with the single plane and multiplanar unenhanced methods, and 1752 with the multiplanar enhanced because for one patient no post-contrast images were available due to an adverse reaction to CM. Using the single plane analyses, 130 BMEs were rated as positive, whereas 46 were rated as equivocal (i.e., 19 areas were rated as “probably no BME” and 27 as “probably BME”); the multiplanar unenhanced analyses increased the confidence in detection in 32 of these areas, whereas the remaining 14 (i.e., 12 in one single young patient) were confidently rated applying CM (Fig. 2). Nine areas confidently rated as positive without the application of CM (i.e., single plane and multiplanar unenhanced method) were not confidently assessable by the post-contrast sequences (i.e., respectively eight BMEs were rated as “probably no BME” and one as “probably BME”, all in patients with at least one other BME confidently diagnosed by all methods). Six BME areas rated as “probably no lesion” on STIR alone were then rated as “no lesion” on both multiplanar analyses (i.e., both, unenhanced and enhanced) (Table 2).

**Table 2:** Diagnostic confidence of bone marrow edema areas detection with the three applied methods and comparison of the percentages of confidence rating

Method	Region-based rating according to a diagnostic confidence 4-point rating scale					
	Definitely no BME (*n*)	Probably no BME (*n*)	Probably BME (*n*)	Definitely BME (*n*)	Examined areas^a^ (*n*)	Certain rating (%)
Single plane method	1576	19	27	130	1752	97.37
Multiplanar unenhanced method	1582	2	12	156	1752	99.2
Multiplanar enhanced method	1584	8	1	159	1752	99.49

	Post hoc Bonferroni-corrected McNemar
Single plane method vs multiplanar unenhanced method	*p* < 0.001
Single plane method vs multiplanar enhanced method	*p* < 0.001
Multiplanar unenhanced method vs multiplanar enhanced method	*p* = 0.405

*BME* area of bone marrow edema

^a^1752 areas were used for comparisons because post-contrast images were not acquired in one patient due to adverse reaction to contrast medium

The percentages of confident ratings for detection obtained with each method were 97.37% for the single plane, 99.20% for the multiplanar unenhanced and 99.49% for the multiplanar enhanced. The Cochran *Q* test revealed a statistically significant difference among the three methods (*p* < 0.001). The post hoc Bonferroni-corrected McNemar test showed, for the confidence in detection, a significant difference between STIR and multiplanar enhanced and unenhanced analyses, respectively (*p* < 0.001, each), but no statistically significant difference emerged between the two multiplanar methods (i.e., with and without contrast) (*p* = 0.405) (Table 2).

The multiplanar unenhanced and enhanced methods increased the confidence in BME detection in at least one area of the SIJ in, respectively, 25 and 27 patients (*p* = 0.219).

The second evaluation of the dataset (i.e., 240 areas) demonstrated the same rating in 218 areas (90.83% of agreement) using only one plane, in 223 (92.91% of agreement) and in 225 (93.75% of agreement) areas using the multiplanar unenhanced and enhanced method, respectively.

### Diagnostic confidence in localization of BME

**Fig. 3:**
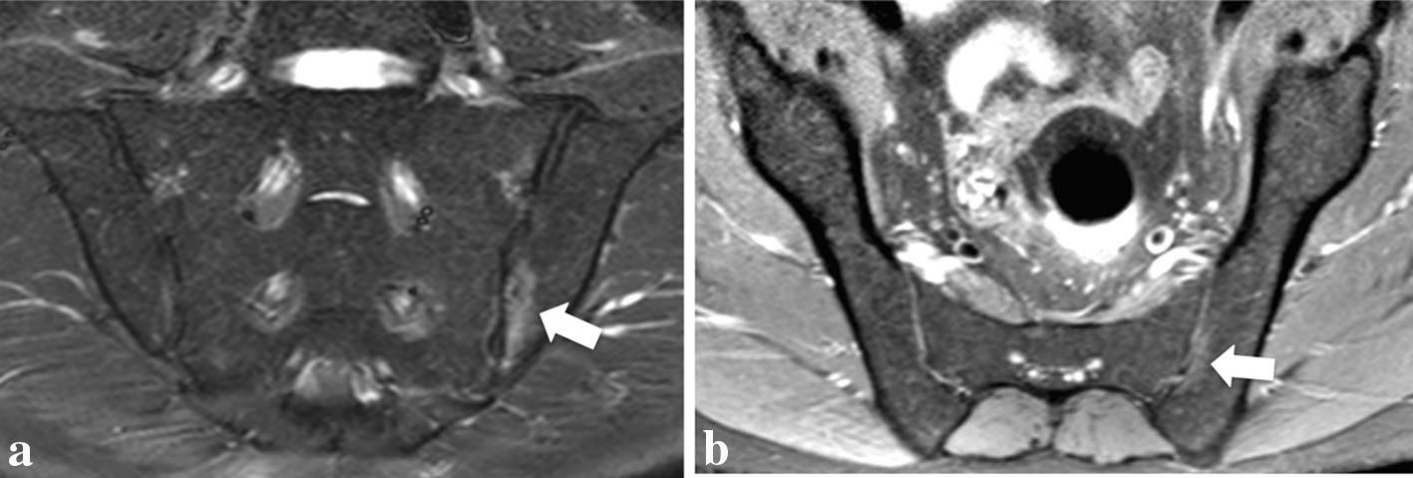
A 39-year-old male patient with spondyloarthritis. Low signal intensity (i.e., SI = 1) area of bone marrow edema in the *left* iliac bone (*white arrows*) confidently detected (i.e., detection’s diagnostic confidence = 4), but with an uncertain rating for localization (i.e., localization’s diagnostic confidence = 3) with the single-plane method (i.e., para-coronal STIR only in **a**). The multiplanar unenhanced method (para-coronal STIR in **a** and para-axial PD-fs in **b**) increased the confidence in localization, allowing a confident localization of this BME area close to the transition point between the anterior and posterior portion of the mid portion of the iliac bone

Twenty-one areas demonstrated equivocal confidence in localization on STIR (i.e., single plane method): three were rated as “probably unable to be localized” and 18 as “probably able to be localized”; none was rated as “unable to be localized” (Fig. 3). Twenty of these 21 BME areas were confidently localized with the multiplanar methods (i.e., both unenhanced and enhanced). One area considered “probably unable to be localized” was not confirmed on either multiplanar unenhanced or enhanced images; therefore, its localization with the last two methods was not feasible. As aforementioned, six areas (i.e., in five patients) were detected only on STIR alone (i.e., detection’s diagnostic confidence = 2) and not on multiplanar methods (i.e., detection’s diagnostic confidence = 1), and therefore, they were also not localized by these last methods.

The Friedman test, applied to compare the confidence in localization for each lesion, showed a significant difference among the three methods. The post hoc Bonferroni-corrected Wilcoxon test showed a statistically significant difference between STIR alone and both multiplanar methods (i.e., unenhanced and enhanced; *p* < 0.001 each), but no difference emerged between the two multiplanar analyses (*p* = 1.00).

Both multiplanar methods showed higher confidence in localization than STIR alone and increased the confidence in 13 patients, demonstrating at least one area of the SIJ with an uncertain rating with the single plane method (i.e., localization’s diagnostic confidence <4).

Independently from the applied methods, most of the identified areas of bone marrow edema (67.1%) were localized in the lower (i.e., middle or inferior) portions of the SIJ.

### Size and signal intensity

The distribution of certain and uncertain rating for detection and localization based on size and SI of the BMEs, and a statistical summary of the reckoned data are shown in Table 3.

**Table 3:** Distribution of certain and uncertain detection and localization diagnostic confidence according to size and signal intensity of the bone marrow edema areas

Size of bone marrow edema areas^a^				
	<1 cm (*n*)	1 ≥ cm < 2 (*n*)	≥2 cm (*n*)	Chi-squared test
Confident detection^b^	62	50	18	*p* = 0.006
Uncertain detection^c^	30	16	0	

				Fisher’s test
Confident localization^d^	78	60	17	*p* = 0.452
Uncertain localization^e^	14	6	1	

Signal intensity of bone marrow edema areas^f^				
	Low grade (*n*)	Intermediate grade (*n*)	High grade (*n*)	Chi-squared test
Confident detection^b^	40	38	52	*p* < 0.001
Uncertain detection^c^	39	7	0	

				Fisher’s test
Confident localization^d^	70	38	47	*p* = 0.694
Uncertain localization^e^	9	7	5	

^a^Assessment was performed on para-coronal STIR and size was measured in the cranio-caudal direction; overall 1764 areas (74 patients) were available for the analyses and 1588 turned out to be negative on para-coronal STIR alone

^b^ = Confident rating for detection included “definitively no BME” and “definitively BME” ratings

^c^ = Uncertain rating for detection included “probably no BME” and “probably BME”

^d^ = Confident rating for localization included “able to localize with confidence”

^e^ = Uncertain rating for localization included “probably unable to localize” and “probably able to localize”

^f^ = Signal intensity of each BME was compared with the signal intensity of spinal fluid

On STIR alone, 52 of the 130 lesions with a confident pathologic rating (i.e., detection’s diagnostic confidence = 4) showed high-grade SI (i.e., grade = 3) and 68 had a cranio-caudal diameter equal to or greater than 1 cm.

Among the 46 regions with an equivocal detection’s confidence using the single plane method (i.e., detection’s diagnostic confidence = 2 or 3), none demonstrated a high-grade SI, whereas 39 showed a low SI and seven an intermediate one; furthermore, no BME area had a cranio-caudal diameter greater than 2 cm and most (i.e., 30 BME areas) were smaller than 1 cm. Thus, a statistically significant difference between the distributions of certain and uncertain detection’s confidence based on the size and SI of BME areas emerged (*p* = 0.006 and *p* < 0.001, respectively).

On STIR alone, among the BME areas with a confidently positive localization’s confidence rating (i.e., localization’s diagnostic confidence = 4), 85 showed either mid- or high-grade SI (i.e., either grade 2 or 3) and 78 had a maximum cranio-caudal diameter less than 1 cm and 77 were equal to or greater than 1 cm.

Among the 21 regions with an uncertain rating using a single plane (i.e., localization’s diagnostic confidence = 2 and 3), 16 showed either low or intermediate grade SI and five showed high-grade SI; furthermore, only one BME area had a cranio-caudal diameter greater than 2 cm and most (i.e., 14 BME areas) were smaller than 1 cm. For the localization, no statistically significant difference emerged between the distribution of certain and uncertain rating based on the size and SI of BME areas (*p* = 0.452 and *p* = 0.694, respectively).

## Discussion

**Fig. 4:**
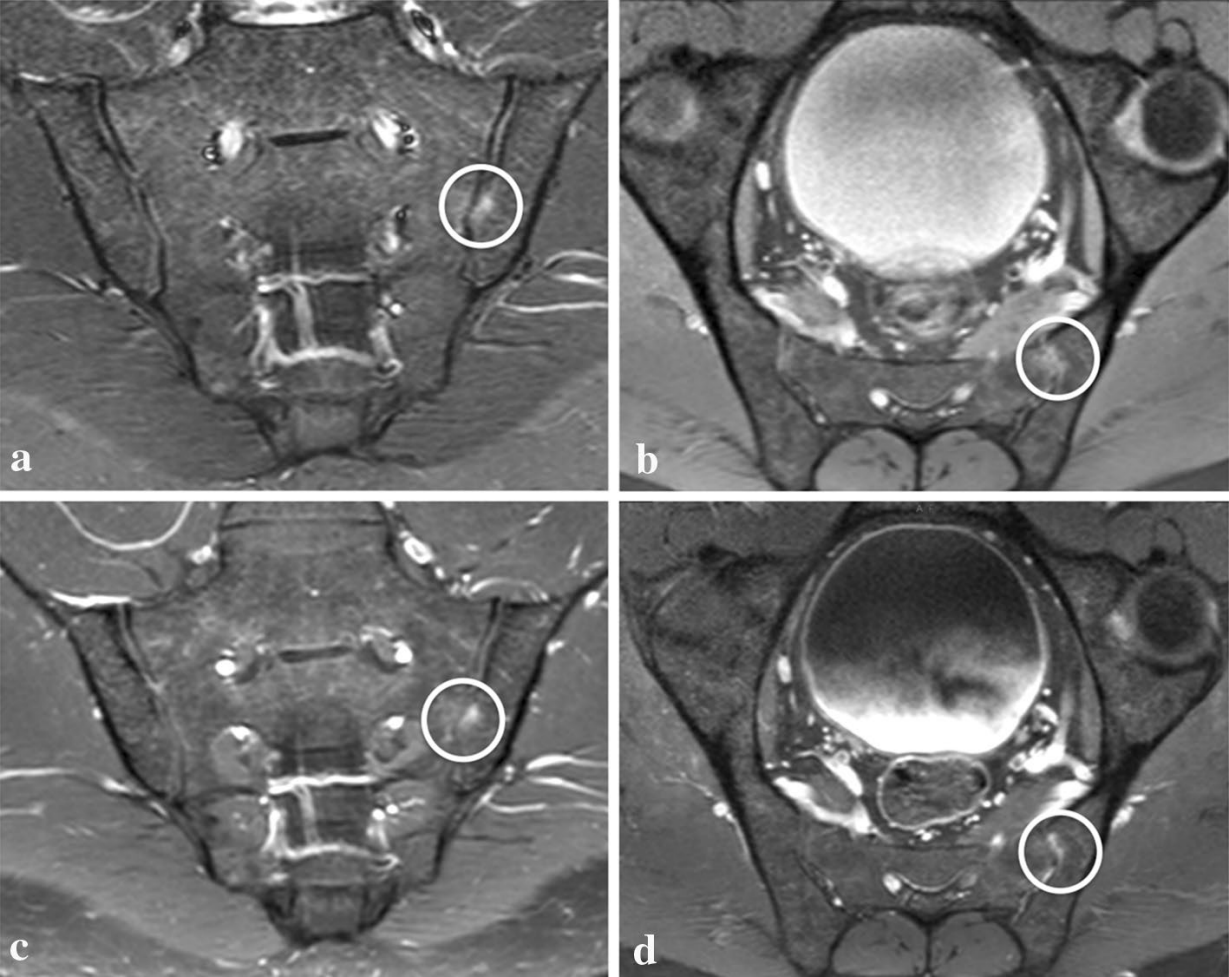
A 27-year-old male patient with spondyloarthritis. Area of bone marrow edema (*white circle*) easily diagnosed (i.e., detection’s diagnostic confidence = 4) and localized (i.e., localization’s diagnostic confidence = 4) with all three methods: single plane method (para-coronal STIR only in **a**); multiplanar unenhanced method (para-coronal STIR **a** and para-axial PD-fs in **b**); and multiplanar enhanced method (para-coronal post-contrast T1-fs in **c** and para-axial post-contrast T1-fs in **d**)

Sacroiliitis is one of the hallmarks of SpA [4, 14], and the assessment of subchondral BME on MRI is essential for early diagnosis and follow-up. An increasing number of studies have emphasized that the presence of subchondral BME, seen on STIR, is considered sufficient to diagnose active sacroiliitis [4, 15, 16], and suggesting that CM is not mandatory and should only be applied in equivocal cases. Althoff et al. [7] declared that the administration of CM might be beneficial by providing a different view of the lesions with another sequence, and when MRI is interpreted by inexperienced readers, or when minimal changes occur. In agreement with the suggestions of Althoff et al. our results showed that the benefits provided not only by a different pulsed sequence, but also by multiple acquisition planes (e.g., para-axial) increase the diagnostic confidence for detecting and localizing each BME area. Thus, in our opinion, the approach for a reliable assessment of BME in doubtful cases, does not rely necessarily on the administration of CM. Indeed, in our population, the multiplanar unenhanced method allowed a high diagnostic confidence in detection and localization in 46 and 20 areas, respectively, which showed a doubtful rating using the single plane method (i.e., detection’s and localization’s diagnostic confidence increased overall in 25 and 13 patients, respectively). Moreover, applying a region-based assessment, no statistically significant difference emerged between the multiplanar methods for either detection or localization percentages of confidence ratings (Fig. 4). Gadolinium has been determinant to reach a high rating in 14 regions, 12 of them in one young patient. Even if some ultimate scientific evidence [17–19] affirm that CM might not be necessary for diagnosing BME in young patients with SpA (i.e., juvenile spondyloarthritis), in our case, it allowed a clear distinction of the increased vascularity [20] and allowed the diagnosis, through pathologic enhancement, of BME otherwise not confidently diagnosable with STIR alone or in association with the para-axial PD-fs. Conversely, in adults with chronic signs and also possibly under treatment, the assessment of BME might become more difficult due to the presence and overlap of areas of inflammation with zones of fat replacement. However, in this setting, STIR has already demonstrated the ability to accurately depict BME even better than after CM application [6, 13, 21]. The same observations were made in our study, where the nine areas with low diagnostic confidence for BME detection on multiplanar contrast images and high rating on unenhanced images (i.e., both STIR alone and multiplanar unenhanced) were close to fat replacement zones. The high SI provided by STIR and its homogenous fat suppression [22, 23] might be considered the main reason for its better performance. Signal intensity and size of the BME areas demonstrated, in our population, to have a role in the confidence of BME detection, as shown by the statistically significant difference in the distribution of certain and uncertain rating according to these two variables (i.e., both assessed on STIR). This evidence and the absence of BME with a cranio-caudal diameter greater than 2 cm and with very high SI among those with uncertain rating let us assume that small areas with low SI benefited from a multiplanar approach for confident detection. As mentioned above, conversely, no statistically significant difference emerged between the distribution of certain and uncertain localization’s rating when size and SI were taken into account: it is then reasonable to assume that uncertain localization is due to the complex anatomical structure of the SIJ more than depending on the SI and the size of BME area, an assumption that might also justify the use of a multiplanar approach. The fact that 67.1% of the areas of BME were located in the lower portion of the sacroiliac joints supports the theory that inflammatory changes are rather present in the lower portions in contrast to overuse BME, which is typically seen in the upper portions.

Further studies that would include a larger population and the investigation of other features of this chameleon-like disease are necessary to fully assess all these findings and to evaluate whether a multiplanar unenhanced method (i.e., STIR and paPD-fs) might also increase the diagnostic confidence for the other SpA features (e.g., synovitis, enthesitis, capsulitis).

## Limitations

The main limitation of this study is that there was only one group of raters. We believe, however, that the presented results have a sufficient reliability and impact because the raters were two experienced and dedicated musculoskeletal radiologists working in consensus and analyzing each dataset (i.e., single plane, multiplanar unenhanced, multiplanar enhanced) with an interval of 4 weeks in-between. Thus, we expect that the application of a multiplanar unenhanced method in the daily clinical setting would have an even higher positive impact on radiologists who are not sub-specialized in the musculoskeletal field.

As this is a retrospective study, the distribution of the clinical and laboratory information, beside the clinical suspect of spondyloarthritis, was not homogeneous; therefore, no correlation between the radiological analyses and the clinical parameters confirming the diagnosis was performed. A future prospective longitudinal study may focus on these aspects and further assess the advantages of the hereby-proposed method.

The inclusion only of patients with clinical suspect of SpA might be considered prone to an overrating of BME and, in association with the absence of controls, to a biasing effect on the readers, but the detection of 37 patients completely negative for sacroiliitis (i.e., absence of any BME with each method) indicates that 50% of the population can be considered as a control group for BME and highlights the accuracy and objectivity of the study.

Size and SI were assessed only on STIR and not on the other sequences, because a quantitative evaluation of the measurements obtained from each set of images could have revealed differences that, presumably, would have been mainly dependent on the intrinsic technical differences (e.g., SNR, fluid sensitivity) of the applied sequences (i.e., STIR, PD-fs, postcontrast T1w fs) [22–25]. A qualitative and quantitative assessment and comparison of the datasets derived from the different sequences based on size and SI of each BME area would have exceeded the aim of this study.

Last, the applied method for the patient-based assessment (i.e., in each patient, the confidence of detection and localization was considered improved when a method allowed an increase in rating in at least one area) might be considered prone to overestimation of the multiplanar methods. However, the correct assessment of each BME has an impact on the therapeutic management at diagnosis as much as during the follow-ups especially for patients affected by SpA. Therefore, we believe that our evaluation strategy is justified.

In conclusion, the application of a multiplanar method can improve the diagnostic confidence in detecting and accurately localizing areas of BME. The absence of statistically significant differences between the proposed unenhanced method, including para-coronal STIR and para-axial PD-fs, and the multiplanar post-contrast method suggests that CM does not provide any additional value for a higher diagnostic confidence. Thus, the application of CM might be further reduced for the assessment of BME in SpA patients.
